# Neonatal exposure to hyperoxia leads to persistent disturbances in pulmonary histone signatures associated with NOS3 and STAT3 in a mouse model

**DOI:** 10.1186/s13148-018-0469-0

**Published:** 2018-03-20

**Authors:** Cho-Ming Chao, Rhea van den Bruck, Samantha Lork, Janica Merkle, Laura Krampen, Patrick P Weil, Malik Aydin, Saverio Bellusci, Andreas C. Jenke, Jan Postberg

**Affiliations:** 1 Excellence Cluster Cardio-Pulmonary System (ECCPS), Member of the German Center for Lung Research (DZL), Department of Internal Medicine II, Universities of Giessen and Marburg Lung Center (UGMLC), Aulweg 130, 35392 Giessen, Germany; 2 University Children’s Hospital Gießen, Division of General Pediatrics and Neonatology, Justus-Liebig-University, Gießen, Germany; 3 Department of Pediatrics, HELIOS Medical Center Wuppertal, Center for Clinical & Translational Research (CCTR), Center for Biomedical Education & Research (ZBAF), Witten/Herdecke University, Wuppertal, Germany; 4 EKO Children’s Hospital, Oberhausen, Witten/Herdecke University, Alfred-Herrhausen Str. 40, Witten, Germany

## Abstract

**Background:**

Early pulmonary oxygen exposure is one of the most important factors implicated in the development of bronchopulmonary dysplasia (BPD).

**Methods:**

Here, we analyzed short- and long-term effects of neonatal hyperoxia on NOS3 and STAT3 expression and corresponding epigenetic signatures using a hyperoxia-based mouse model of BPD.

**Results:**

Early hyperoxia exposure led to a significant increase in NOS3 (median fold change × 2.37, IQR 1.54–3.68) and STAT3 (median fold change × 2.83, IQR 2.21–3.88) mRNA levels in pulmonary endothelial cells with corresponding changes in histone modification patterns such as H2aZac and H3K9ac hyperacetylation at the respective gene loci. No complete restoration in histone signatures at these loci was observed, and responsivity to later hyperoxia was altered in mouse lungs. In vitro, histone signatures in human aortic endothelial cells (HAEC) remained altered for several weeks after an initial long-term exposure to trichostatin A. This was associated with a substantial increase in baseline eNOS (median 27.2, IQR 22.3–35.6) and STAT3α (median 5.8, IQR 4.8–7.3) mRNA levels with a subsequent significant reduction in eNOS expression upon exposure to hypoxia.

**Conclusions:**

Early hyperoxia induced permanent changes in histones signatures at the NOS3 and STAT3 gene locus might partly explain the altered vascular response patterns in children with BPD.

## Background

Bronchopulmonary dysplasia (BPD) is a chronic lung disease of prematurely born infants and remains a leading cause of morbidity and mortality. Currently, there is no curative therapy available. Based on the severity-based definition of BPD (inclusion of infants with mild BPD), 68% of premature infants born with a gestational age (GA) ≤ 28 weeks develop BPD [1–3]. The risk of developing BPD correlates inversely with the gestational age (GA) and birth weight (BW) [4]. Since premature infants are born with a lung which is in the canalicular or saccular stages of development, the lung structure is therefore not adequate to provide sufficient ventilation and gas exchange. Thus, mechanical ventilation and high oxygen concentration are often necessary at birth. Barotrauma induced by mechanical ventilation as well as oxygen toxicity and inflammation are major contributing factors responsible for the pulmonary damages in the immature lung. In addition, some studies have suggested a strong genetic component in BPD [5]. In fact, clinical studies suggest that decisions during the first minutes of life [6] or even events before the delivery might be crucial for the later development of BPD. In line with these clinical data are observations that hyperoxia in rat pups leads to increased endothelilal nitric oxide synthase (eNOS) levels, nitric oxide (NO) activity, hyperemia [7, 8], and possibly eNOS uncoupling eventually leading to BPD.

Importantly, BPD does not only affect neonatal mortality but also leads to long-term morbidity, e.g., increased susceptibility to upper respiratory infections during the first year of life [9] and pulmonary hypertension [10]. Again, neonatal oxygen seems to be an important pathological factor since it increases for example the sensitivity to influenza A virus infection by suppressing epithelial expression of Ear1 [11]. In fact, extremely and moderately preterm infants face a 3.6 times increased risk of being hospitalized due to respiratory infection in the first year of life [12].

Whereas it seems clear from twin studies that genetic susceptibility plays an important role for disease development [13], the exact mechanism remains unclear but epigenetic mechanisms seem to be part of it since several recent publications have reported abnormalities of histone acetylase activity and the chromatin remodeling pathway in BPD patients [14, 15]. Thus, persistent changes in epigenetic signatures might be also at least partly responsible for the later development of pulmonary hypertension and the increased susceptibility to respiratory tract infections. Since we have recently shown that epigenetic signatures at NOS3 encoding eNOS in human umbilical artery endothelial cells are substantially shaped by prenatal events such as placental insufficiency and that there exists an interdependency between NOS3 and the gene activity of signal transducer and activator of transcription 3 alpha (STAT3α) as well as Stat3-transcription factor-binding in the NOS 3 promoter. Moreover, in human, a 27-nt non-coding RNA becomes co-expressed with NOS3 and post-transcriptionally regulates STAT3 expression [16]. We thus aimed to utilize the same gene loci to analyze the effect of early neonatal oxygen exposure on the pulmonary vascular endothelial epigenetic landscape and the associated consequences for oxygen exposure later in life.

Therefore, we analyzed NOS3 gene expression in response to early neonatal hyperoxia at first and then studied the respective changes in epigenetic signatures as consequences of this first early oxygen exposure on later events of hyperoxia and hypoxia later in life.

## Methods

### Mice

Wildtype mice (males and females, CD1 background) were crossed to generate time-pregnant females. All mice received food and water ad libitum.

### Hyperoxia injury (BPD mouse model)

**Fig. 1:**
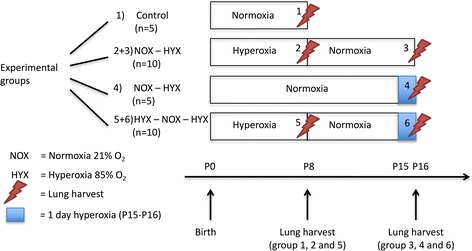
Experimental setting. The experimental setting consists of six groups. Each group consists of five animals. The experiments were performed in two steps. The first comprised a control group at P8 in normoxia (group 1), a group after neonatal hyperoxia from P0 to P8 (group 2), and a group exposed to neonatal hyperoxia from P0 to P8 with eight additional days of normoxia until P16 (group 3). The second comprised a control group in normoxia at P16 (group 4), a group after neonatal hyperoxia from P0 to P8 (group 5), and a group exposed to neonatal hyperoxia (P0 to P8) followed by normoxia (P8 to P15), and a second exposure to hyperoxia for 1 day before analysis at P16 (group 6). P postnatal

Newborn pups were exposed to either normoxia (NOX) or hyperoxia (HOX) within 24 h after birth (P0) (Fig. 1). In experimental group 1, dams and pups were kept in NOX from P0 to P16. In experimental group 2, dams and pups were kept in NOX from P0 to P15. From P15 to P16, the dams and the pups were exposed to HOX (85% O_2_). In experimental group 3, newborn pups were subjected to HOX (85% O_2_) injury from P0 to P8 in a chamber (Proox Model 110, Biospherix). To minimize oxygen toxicity and bias, nursing dams were rotated every 24 h between NOX and HOX. Afterwards, nursing dams and pups were exposed to NOX (21% O_2_) from P8 to P16. From P15 to P16, the dams and the pups were re-exposed to HOX (85% O_2_). In experimental groups 1 and 3, lungs were harvested at P8. In all groups, lungs were harvested at P16. All dams and pups received food and water ad libitum.

### Lung perfusion, isolation, tissue processing, and histology

Mice were euthanized by intraperitoneal injection of a solution made of ketamin, dormitor, heparin, and saline. After sternotomy, the lung was perfused transcardially (through the right ventricle) by using PBS (1×), then isolated and incubated for 30 min at 4 °C in COLD medium, stored at − 20 °C overnight, and finally stored at − 80 °C till further tissue processing.

For histology, the lung was flushed from the right ventricle to remove blood cells then perfused through the trachea with a pressure of 20 cm H_2_O with 5 ml 4% PFA. The trachea was tied off with a string, and the lung was removed and placed in 4% PFA for max. 24 h at 4 °C. Lungs were then progressively dehydrated (30, 50, 70, and 99% ethanol, 3 h each), incubated in xylole, then in paraffine overnight and finally embedded with a Leica embedding machine (EG 1150C). Paraffin blocks were kept cold, and 5 μm sections were cutted. Hematoxylin and eosin staining was performed according to protocols previously published.

### Positive selection for vascular endothelial cells

Positive selection for vascular endothelial cells was performed by magnetic separation with human CD146 Microbeads (Miltenyi®, Bergisch-Gladbach, Germany) following manufacturers’ instructions with minor modifications as described previously [17].

### RNA isolation

Total RNA was isolated using Trizol (Sigma-Aldrich) and isopropanol precipitation and further purification on columns. Next, RNA integrity was assayed using the Agilent Bioanalyzer 2000. Only samples with non-fragmented RNA were included.

### Gene expression analyses

Gene expression analyses were performed using quantitative real-time PCR (qPCR) analyses on a Rotor-Gene 6000 (Qiagen). For PCR reactions, QuantiTect SYBR Green qPCR Master Mix (Qiagen) containing Hot Start Taq DNA polymerase and SYBR Green was used. Primers were used as described earlier [18]. The expression of gene of interest was normalized using three housekeeping genes (BACT, GAPDH, PECAM1). PCR conditions were as follows: 95 °C for 15 min, 40× [95 °C for 15 s, 60 °C for 30 s]. Melting of PCR product was done using a gradient from 55 to 95 °C rising in 0.5 °C increments.

### Antibodies

Primary antibodies used in this study were (1) Rabbit anti-H2A.Zac (Diagenode pAb-173-050), (2) Rabbit anti-H3K9ac (Active Motif pAb#39137), and (3) Rabbit anti-H3K4me3 (Diagenode pAbCSP-030-050).

### Chromatin purification and ChIP assays

Chromation purification and ChIP assays were performed as described previously [15]. Quantitative PCR analyses were performed using a Rotorgene 6000 (Qiagen). The relative amounts of specifically immunoprecipitated DNA were estimated as “percent of input” and quantified using individual standard curves for each amplicon. Primer pairs were used as described earlier [18].

### Pyrosequencing

DNA methylation signatures of promoter segments of NANOG, NFE2L2, and STAT3 were analyzed using a Qiagen Pyromark Q24 sequencer as previously described (Plos One Jenke et al.). Briefly, after standard sodium bisulfite conversion using the EZ DNA Methylation-Gold Kit (Zymo Research, USA) pyrosequencing methylation analysis was conducted using the PyroMark Q24 (Qiagen, Germany) according to the manufacturer’s protocol. Therefore, we designed and made use of the following oligonucleotides: 1. for NFE2L2—primer F1 (5′- gga gtt aga ggg gat agt ggt t-3′), 5′-biotinylated primer (5′-acc cca cca aat caa aac ttc ct-3′), and sequencing primer S1 (5′-agg gta aag gag gat g-3′); 2. for STAT3—primer F1 (5′-ggt gta ggg tgg ggt tat t-3′), 5′-biotinylated primer (5′-acc cta tat atc tcc tcc tat cct-3′), and sequencing primer S1 (5′-ggg tgg ggt tat ttt t-3′); 3. for NANOG (DNA methylation positive control)—primer F1 (5′-gta gga tag gaa tgg ggg ttg-3′), 5′-biotinylated primer (5′-acc tta aat tta ccc caa att cta c-3′), and sequencing primer S1 (5′-aat ggg ggt tgg gga-3′). No reliable NOS3 DNA methylation assays meeting our quality standards could be designed. The level of methylation was analyzed using PyroMark Q24 2.0.6 Software (Qiagen). Non-CpG cytosine residues and a standard fully methylated DNA (Zymo Research, USA) were used as controls to verify bisulfite conversion.

### Small RNA-seq and analyses pipeline

Total RNA was purified as described above. For multiplexing, we made use of different multiplex sequencing barcodes for sequencing in a single lane as described [19]. Briefly, total RNA was separated by polyacrylamide gel electrophoresis. Gel fragments corresponding to 15 to 35 nt RNA molecules were cut, and RNA was eluted. These small RNAs were directly used for the construction of sequencing libraries in four steps: step 1: ligation of DNA oligos to the 3′-end of the RNA; step 2: ligation of RNA or, respectively, chimeric RNA/DNA oligos to the 5′-end of RNAs; step 3: cDNA library synthesis by reverse transcriptase; and step 4: amplification of the cDNA library. Subsequently, after final quality checks by microcapillary electrophoresis and qPCR, the libraries were sequenced on an Illumina Hiseq 2000 platform (single end, 50 bp). This work has benefited from the facilities and expertise of the high-throughput sequencing core facility of IMAGIF Gif-sur-Yvette (Centre de Recherche de Gif—www.imagif.cnrs.fr). The initial data analysis pipeline was as follows: CASAVA-1.8.2 was used for demultiplexing, Fastqc 0.10.1 for read quality assessment and Cutadapt-1.3 for adaptor trimming, resulting in an average sequence number for each developmental time point sample of 7.58 Mbp. File conversions, filtering, and sorting as well as mapping (Bowtie2) were done using “Galaxy” [20–22], a platform for data intensive biomedical research (https://usegalaxy.org/), and “Chimira” [23], respectively.

### Immunofluorescence staining for eNOS

Paraffin sections were deparaffinized. Antigen retrieval was performed for 15 min in citrate buffer using a rice cooker, then slides were cooled down on ice for 20 min. After washing slides three times in TBST (TBS buffer + 0.1% Tween 20) for 5 min, slides were blocked with 3% bovine serum albumin (BSA) and 0.4% Triton X-100 [in Tris-buffered saline (TBS)] at room temperature (RT) for 1 h and then incubated with primary antibody against eNOS (ThermoFisher Scientific, PA1-037; 1:100) at 4 °C overnight. Afterwards, slides were again washed three times in TBST for 5 min, incubated with secondary antibody (Goat Anti-Rabbit Alexa 555, ThermoFisher Scientific, A-21429; 1:500) in room temperature for 1 h, and then washed three times in TBST before being mounted with Prolong Gold Anti-fade Reagent with DAPI (4,6-diamidino-2-phenylindole; ThermoFisher Scientific, P36931). Fluorescent images were acquired using Leica DM5500 B fluorescence microscope connected to Leica DFC360 FX camera.

### Statistical analysis

Data were compared using Mann-Whitney *U* test according to normality assumptions on univariate analysis followed by Bonferroni correction for multivariate analysis. Categorical variables were compared using the Fisher exact test. Statistical analyses were performed with GraphPad Prism 5.0.

### Cell culture

Human arterial endothelial cells (HAEC) were bought from PromoCell (Heidelberg, Germany) and cultivated upon manufacturer’s recommendations. For cDNA synthesis, we used 500 ng RNA per sample using the QuantiTect Reverse Transcription kit (Qiagen). DNA was isolated by phenol:chloroform:isoamylic alcohol extraction followed by precipitation with isopropanol. For in vitro experiments, HAEC (PromoCell) were cultivated upon manufacturer’s recommendations. For HDAC inhibition, HAEC were treated with 1 μM trichostatin A (TSA) for either 72 h or twice for 6 h with an 60-h interval in between. Hypoxia experiments were performed using a hypoxia incubator chamber (STEMCELL Technologies, Grenoble, France) exposing cells to a ppO2 of 0.12 bar corresponding to an oxygen fraction of 12% for 24 h. Control experiments were performed under normoxic conditions (ppO2 = 0.21 bar).

## Results

### Neonatal hyperoxia from P0 to P8 leads to increased NOS3 expression—in both whole lung tissue and CD146-positive cells

**Fig. 2:**
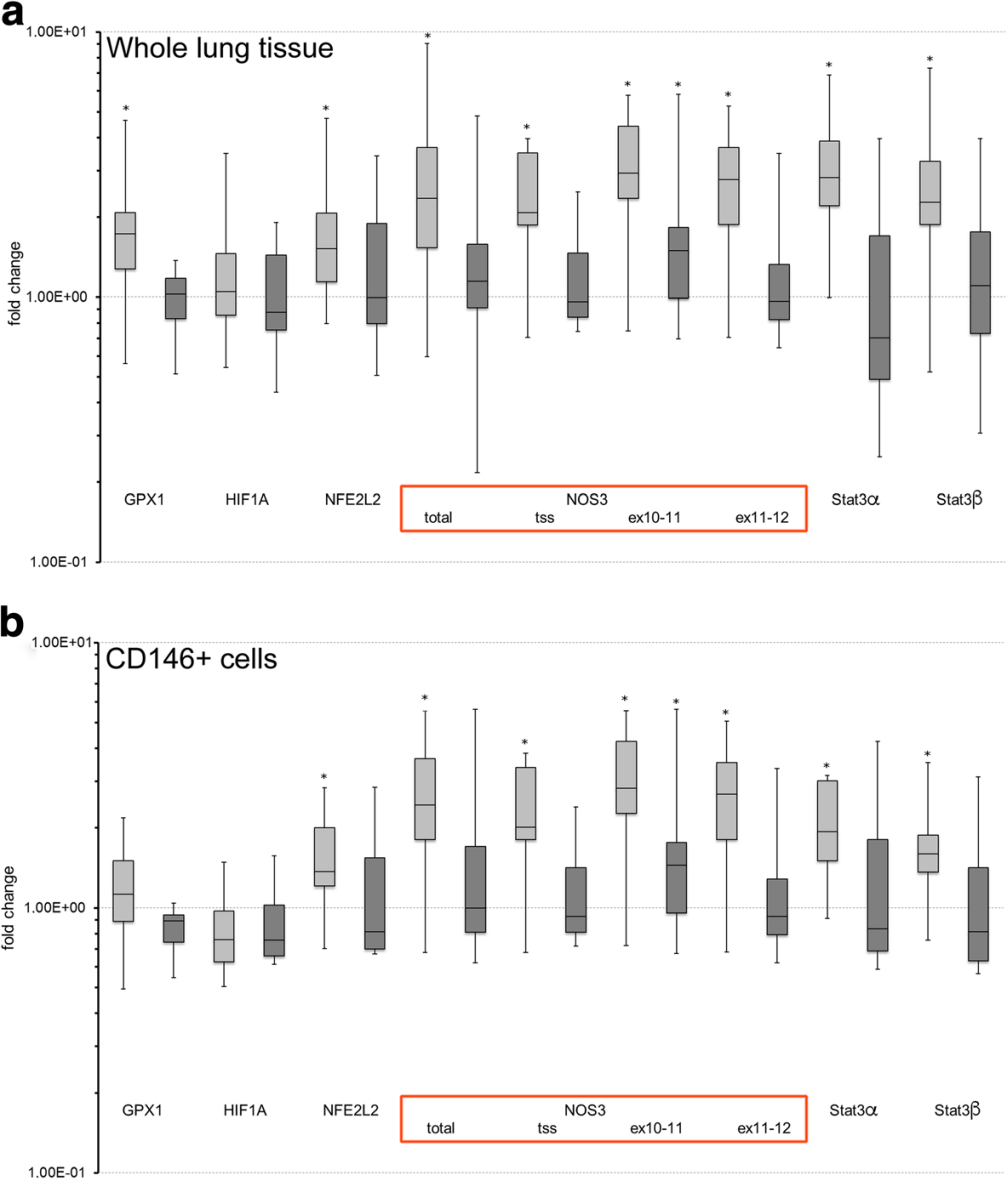
Expression profile of whole lung tissue versus endothelial cells (CD 146+). The upper part shows expression patterns in whole lung tissue (**a**), the lower in CD146-positive endothelial cells (**b**). Light gray bars indicate mice immediately after hyperoxia (group 2), dark gray 8 days of normoxia after hyperoxia from P0 to P8 (group 3). Expression data is normalized to the control group 1. Every group contained five animals. Error bars indicated 95% CI. Asterisks indicate significant difference to the control group (*p* < 0.05)

**Fig. 3:**
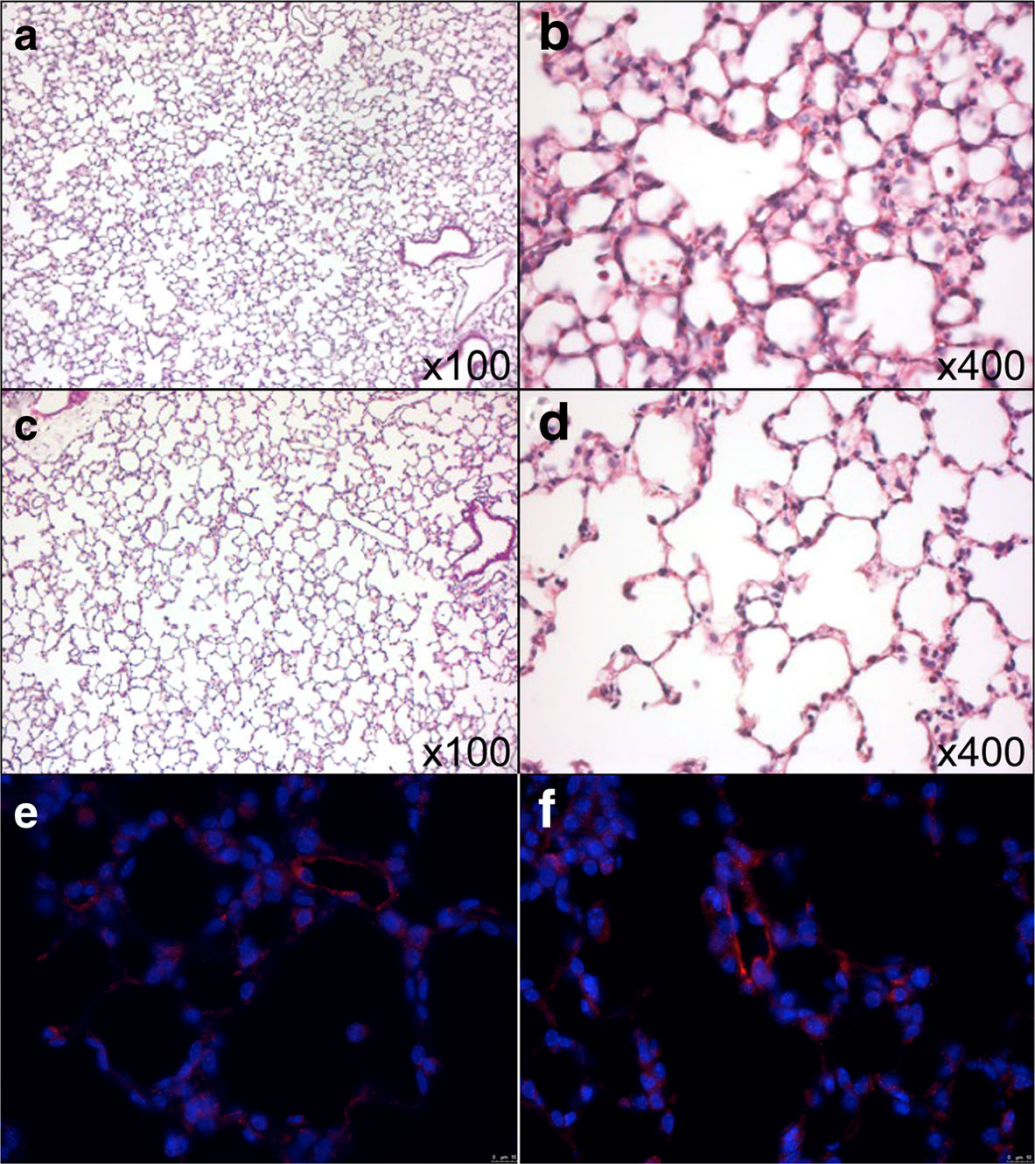
Neonatal hyperoxia leads to hypoalveologenesis at P8. **a**, **b** The lungs of the normoxia group at P8 (group 1) show normal developed alveoli. **c**, **d** The lungs from the hyperoxia group (group 2) at P8 after exposure to 85% oxygen from P0 to P8 reveal a significant hypoalveologenesis with dilated alveoli and increased airspace macroscopically due to disturbed secondary septa formation. H&E. **e**, **f** Immunofluorescence staining on P8 comparing normoxia (**e**, group 1) versus hyperoxia (**f**, group 2) show higher expression after hyperoxia. **a**, **c** × 100; **b**–**f** × 400

For our study, we used a well-established mouse model for bronchopulmonary dysplasia. Newborn pups were exposed to 85% oxygen from P0 to P8 then stayed in normoxia from P8 to P16. Lungs were analyzed at P8 and P16. In neonatal mouse lungs, exposure to hyperoxia (85% oxygen) from P0 to P8 led to a significant increase in NOS3 (median fold change × 2.37, IQR 1.54–3.68, *p* = 0.003), GPX1 (median fold change × 1.73, IQR 1.28–2.08, *p* = 0.001), and STAT3 α and β (median fold change × 2.83, IQR 2.21–3.88, *p* = 0.001) mRNA levels at P8. Other genes, such as HIF1A for example, were not differentially expressed on the mRNA level (Fig. 2a). At P16, 8 days after hyperoxia, mRNA levels of GPX1 and STAT3 dropped to levels comparable to the normoxia group at P8. NOS3 mRNA levels also dropped but tended to be slightly above the level of the normoxia group at P8—even though this difference did not reach statistical significance except for primer pair matching to exon10/11 (Fig. 2a). In addition, we aimed to determine whether expression profiles upon oxygen exposure differ between whole lung tissue and pulmonary endothelial cells. Therefore, we isolated vascular endothelial cells from whole lung tissue using magnetic separation with human CD146 microbeads. Results obtained from CD146-positive cell population are shown in Fig. 2b. For all analyzed genes of interest, we did not see any differences in expression profiles. After hyperoxia, histology showed simplification of alveoli with increased airspace and dilated alveoli (Fig. 3c, d) compared to the normoxia group (Fig. 3a, b) indicating hypoalveologenesis due to disturbed secondary septa formation. This could be associated with the vascular malformation seen in children with BPD [24, 25],

### Responsivity to later hyperoxia at P15 is altered in mouse lungs that have been exposed to hyperoxia in the neonatal period

**Fig. 4:**
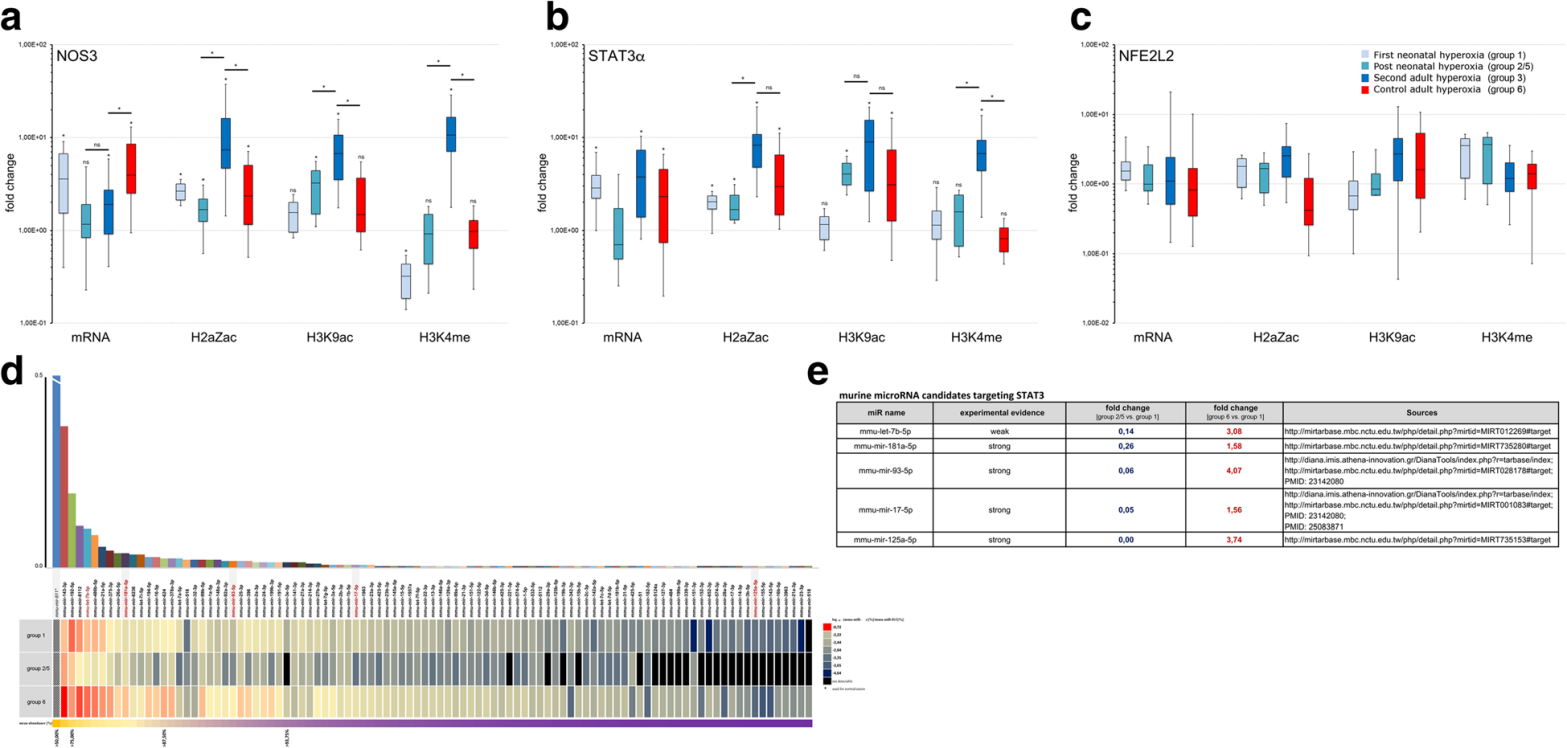
Expression and histone modifications after the first and second period of hyperoxia. **a**–**c** Expression and histone modifications are shown for NOS3, STAT3α, and NFE2L2. Light blue bars represent experimental group 2 (P8, immediately after hyperoxia), medium blue experimental group 3 (P16, 8 days after hyperoxia) and dark blue group 6 (P16, immediately after the second hyperoxia). Red bars represent unexposed adolescent mice after exposure to hyperoxia (group 4). All data is normalized to the control group, i.e., unexposed mice sacrificed at day 7 of life (group 1). Every group contained five animals. Error bars indicated 95% CI. Asterisks indicate significant difference to the control group (*p* < 0.05). **d**, **e** Results of microRNA profiling of group 1 (P8, control group), groups 2 and 5 (P8, immediately after hyperoxia), and group 6 (P16, immediately after the second hyperoxia)

Exposure to hyperoxia later in life at P15 led to substantial differences in mRNA levels compared to mice previously exposed to hyperoxia during the neonatal period and those without. More specifically, there was less increase in NOS3 mRNA levels (median fold change × 1.90, IQR 0.92–2.72) in mice that were previously exposed to hyperoxia compared to mice exposed to normoxia (median fold change × 3.93, IQR 2.50–8.51). Interestingly, the increase in STAT3 mRNA was similar in both groups—even though it tended to be higher in the hyperoxia (median 3.73, IQR 1.38–7.29) compared to the normoxia group (median 2.30, IQR 0.74-4.53)—when compared to the median fold change (× 2.83, IQR 2.21–3.88) after the first exposure. With respect to the other genes investigated, such as NFE2L2 (Fig. 4c), GPX1, and HIF1A (data not shown), we found no significant differences between mice with and without previous hyperoxia exposure (Fig. 4). Since in human, a small 27 nt-RNA is apparently involved in the negative regulation of NOS3 mRNA via STAT3 mRNA targeting, we performed holistic analyses of microRNA (miR) profiles in purified lung epithelial cell obtained from experiments under normoxic (group 1), hyperoxic (groups 2/5), and after repetitive hyperoxic treatment (group 6). Notably, an ortholog of human 27 nt-RNA could not be identified in the mouse genome. Therefore, we aimed to identify differentially expressed murine candidate miRs, which potentially could target STAT3 mRNA in a way reminiscent of human 27 nt-RNA and human STAT3 mRNA. In total, we identified 301 different miRs from which 101 were non-marginally expressed and thus were considered for differential analyses. Figure 4d shows the relative average enrichment of these 101 miRs and below the group-specific quantitative profiles as a heat map. Interestingly, almost general drop of miR abundance was observed after hyperoxic treatment (groups 2/5) when compared with normoxia (group 1), whereas the abundance of many miRs increases markedly, when a repetitive oxygen treatment was applied (group 6). Notably, we identified several miRs differentially overexpressed under these conditions, where strong evidence exists for STAT3 mRNA targeting in mice (Fig. 4e), i.e., mmu-let-7b-5p, mmu-miR-181a-5p, mmu-miR-93-5p, mmu-miR-17-5p, and mmu-miR125a-5p.

### Modifications in histone signatures at the NOS3 and STAT3 gene locus in response to hyperoxia exposure are not restored

We then further analyzed in pulmonary vascular endothelial cells whether changes in histone and/or CpG signatures were associated with the observed expression patterns. Whereas we did not see any significant differences in CpG methylation (Table 1), histone modifications patterns at the NOS3 and STAT3 gene locus changed substantially. Upon initial exposure to hyperoxia, we noted an increase in H2aZac at both loci and an additional decrease in H3K4me3 at the NOS3 locus. No significant alterations were observed for H3k9ac. Importantly, levels for H2aZac did not return to a baseline level but remained elevated and levels for H3K9ac increased significantly after 8 days of normoxia at both loci (Fig. 4a, b). Interestingly, upon second exposure to hyperoxia, we observed an even further increase in H2aZac, H3K4me, and H3K9ac in mice with previous excessive oxygen exposure when compared to mice raised in a normoxic environment, which showed an acetylation/methylation pattern very similar to the initial pattern observed upon the primary excessive oxygen exposure (Fig. 4a, b). At the NFE2L2 locus, no changes were noted (Fig. 4c).

**Table 1:** CpG methylation

	NANOG				NFE2L2						STAT3					
	CpG 1	CpG 2	CpG 3	CpG 4	CpG 1	CpG 2	CpG 3	CpG 4	CpG 5	CpG 6	CpG 1	CpG 2	CpG 3	CpG 4	CpG 5	CpG 6
Ctrl	5.00	10.00	11.50	13.00	3.50	1.00	1.50	3.00	1.50	1.50	2.50	2.00	5.50	1.50	1.50	0.50
Hyperoxia	5.00	11.00	12.00	12.00	3.00	1.00	1.00	2.00	1.00	1.00	3.00	1.00	3.00	1.00	1.00	1.00
*P*[Ctrl/hyperoxia]	0.43	0.47	0.44	0.43	0.14	0.68	0.43	0.09	0.29	0.05	0.48	0.12	0.35	0.86	0.93	0.53
Hyperoxia/5d release	8.00	13.00	12.00	16.00	3.00	1.00	1.00	3.00	1.00	1.00	3.00	2.00	5.00	1.00	1.00	2.00
*P*[Ctrl/hyperoxia-release]	0.80	0.56	0.48	0.71	0.39	1.00	0.79	0.44	0.93	0.28	0.75	0.11	0.89	0.64	0.93	0.18

The mean percentage of methylation at the respective CpGs located in the different promoter regions is shown

### Long-lasting disturbances in histone modification at the NOS3 gene locus lead to disruption of the regulation of eNOS expression upon physiological stimuli later on

**Fig. 5:**
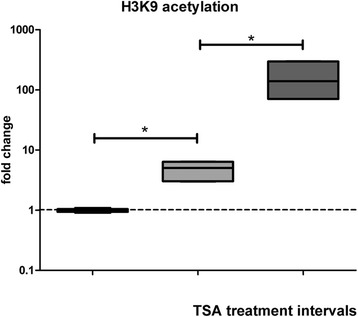
H3K9 acetylation at the NOS3 gene locus after TSA treatment. HAEC were exposed to either trichostatin A (TSA) twice for 6 h with and interval of 60 h (light gray) or once for 72 h. Histone modification was analyzed using CHiP immediately after the TSA exposure and compared to unexposed HAEC (black). All experiments were performed in triplicates. Error bars indicated 95% CI. Asterisks indicate significant differences (*p* < 0.05)

**Fig. 6:**
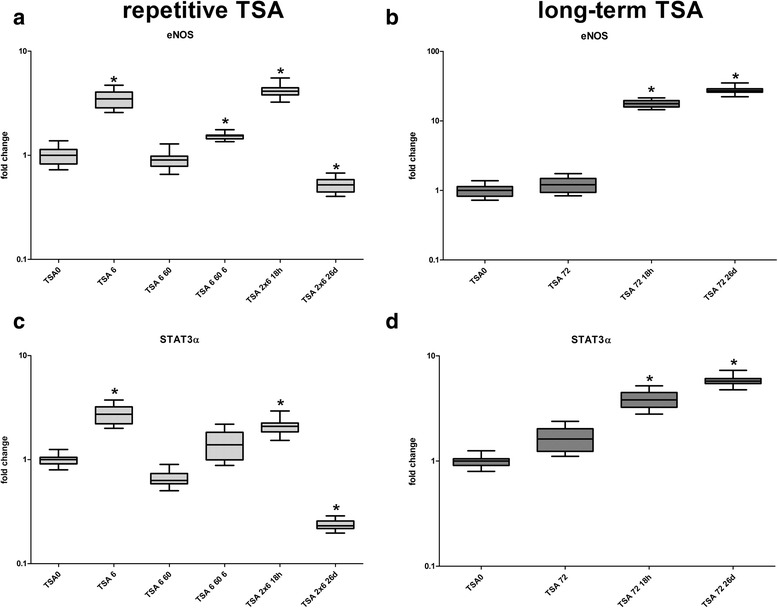
eNOS and STAT3α expression in HAEC after TSA treatment. **a**, **c** HUAEC were treated twice with TSA for 6 h with an interval in between of 60 h. **b**, **d** HAEC were treated once with TSA for 72 h. eNOS and STAT3α expression was analyzed at different time points. All experiments were performed three times with three different HAEC lines. Error bars indicated 95% CI. Asterisks indicate significant differences (*p* < 0.05)

To further analyze whether persistent histone modifications are primarily responsible for the altered eNOS response pattern of vascular endothelial cells upon physiological stimuli later on, we used human aortic endothelial cells (HAEC) which were exposed to trichostatin A (TSA). To mimic persistent environmental stimulation, we incubated HAEC with TSA for 72 h and compared these to unexposed HAEC and HAEC that were exposed twice for 6 h with an interval of 60 h in between to simulate a more physiological situation. Whereas persistent stimulation led to a change in histone acetylation as for example at H3K9, this effect was much weaker in HAEC subjected to repetitive stimulation (Fig. 5). Importantly, we also noticed significant differences between both groups in eNOS and STAT3α expression. Whereas after repetitive stimulation eNOS (median 0.52, 0.4–0.67) and STAT3α (median 0.23, 0.2-0.29) dropped to levels below baseline measurement levels after 4 weeks (Fig. 6a, c), we noticed a substantial increase in baseline eNOS (median 27.2, 22.3–35.6) and STAT3α (median 5.8, 4.8–7.3) expression in HAEC initially subjected to long-term TSA treatment (Fig. 6b, d). Interestingly, the drop to baseline expression of eNOS and STAT3α in the group with repetitive stimulation took significantly more time compared with the first stimulus.

**Fig. 7:**
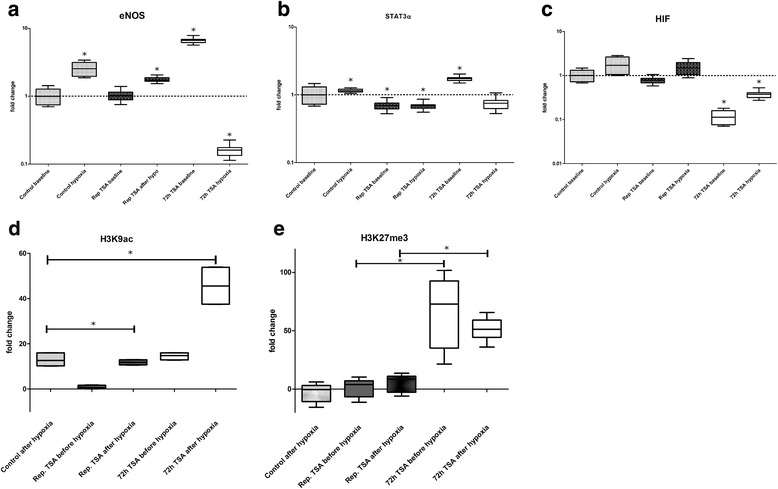
Response to hypoxia after previous treatment with TSA. **a** eNOS expression. **b** STAT3α expression. **c** HIF (hypoxia inducible factor) expression. **d** H3K9ac. **e** H3K27me3. HAEC were either treated with TSA twice for 6 h with an interval of 60 h (light gray) or once for 72 h (white). After culture under normal conditions for 26 days, cells were then subjected to hypoxia (ppO_2_ of 0.1 bar for 24 h). Histone modifications were analyzed at the promoter region. All experiments were performed three times with three different HAEC lines. Error bars indicated 95% CI. Asterisks indicate significant differences (*p* < 0.05)

In the last part of this study, we analyzed the response of the TSA-exposed HAEC to a physiological stimulus. Since in contrast to pulmonary vascular endothelial cells, eNOS expression in peripheral arterial endothelial cells is stimulated by hypoxia, HAEC—either exposed to two 6-h treatments with TSA or one 72-h TSA treatment—were exposed to hypoxia after cultivation for 3 weeks under normal growth conditions. As can be seen in Fig. 7, HAEC with a previous long-term exposure to TSA did show a pathological response to hypoxia with a significant reduction in eNOS expression whereas cells with a previous short-term repetitive exposure to TSA showed a response pattern very similar to control HAEC. This pathological response was accompanied by altered histone modifications at the NOS3 gene locus (Fig. 7d, e). Importantly, as previously mentioned, HAEC with previous long-term exposure to TSA showed persistence of H3K9ac at the NOS3 gene locus whereas in cells with short-term repetitive TSA treatment histone marks at the NOS3 gene locus were restored. Interestingly, we also noticed high levels of the repressive histone mark H3K27me3 which persisted during hypoxia.

## Discussion

Bronchopulmonary dysplasia (BPD) remains one of the main problems of prematurity even in the post-surfactant area. Importantly, BPD is also the main reason for pulmonary hypertension in the pediatric population. Early oxygen exposure has been identified as one of the most important risk factors for the development of BPD. Even though the exact mechanism remains unclear, it seems to involve high oxygen exposure possibly by inducing excessive pulmonary vasodilatation and production of oxygen radicals followed by tissue damage. However, to date, no pathophysiological link has been identified between early oxygen exposure and later pathological response patterns of the pulmonary vasculature in pulmonary hypertension.

Here, we provide the first evidence that excessive oxygen exposure early in life leads to permanent changes in the epigenetic signatures at the NOS3 locus in pulmonary endothelial artery cells in a mouse model. These changes seem to be associated with a non-physiological expression pattern of eNOS upon oxygen exposure later in life. Moreover, non-physiological alterations of epigenetic signatures at the NOS3 gene locus induce similar effects in human aortic endothelial cells (HAEC) in vitro.

Specifically, in the first part of the study, we demonstrated that NOS3 mRNA levels increased in pulmonary artery cells upon neonatal oxygen exposure in newborn mouse. This was paralleled by hyperemia and later disruption of the alveolar structure as shown by histology. This is in line with previous reports demonstrating increased protein concentrations [7] and increased NOS3 mRNA levels upon early excessive oxygen exposure in various models [26] and human tissue samples [27]. Interestingly, we did not observe any changes in RNA expression of NFE2L2, a transcription factor that regulates the expression of antioxidant proteins that protect against oxidative damage triggered by injury and inflammation. This fits to the previous observation that SOD3, an Nrf2-independent antioxidant, was not found to be upregulated as one might expect in the O_2_-exposed neonatal mice compared with room air [28]. In fact, this possibly reflects the inefficiency of counteractive antioxidant mechanisms in the neonatal lung.

The observed increase of NOS3 mRNA levels was paralleled by an increase in H3K9 and H2Az acetylation very similar to our previous observations in HUAEC [18]. Importantly, we did not observe a restitution of acetylation patterns at the NOS3 gene locus after 5 days of normoxia. Moreover, upon a second exposure to hyperoxia later in life, we noticed a dramatic increase in H3K9 and H2Az acetylation and also H3K4 methylation in mice that have been exposed to neonatal hyperoxia. However, NOS3 mRNA levels did not increase but were even found to be substantial lower in these mice when compared to healthy controls. Obviously, the substantial increase in activating histone marks at the NOS3 gene locus did not lead to an increase in transcription in these mice. This is in line with a previous report demonstrating a suppressive effect on NOS3 mRNA synthesis after 12 h incubation with TSA [29]. As we have previously shown the most reasonable explanation for this phenomenon is an increased activity of counteracting processes mainly due to a negative feedback loop, which in humans presumably involves a co-processed 27 nt-ncRNA encoded in NOS3 intron 5 [16, 30], which targets and suppresses STAT3 mRNA [31]. Our analyses allow us to speculate that a negative regulatory function in mice, where a STAT3-targeting 27 nt-RNA was not identified, could be fulfilled by one or several of the differentially expressed miRs, which we identified by microRNA expression profiling.

Moreover, we also observed increased levels of H3K27me3—a repressive histone mark—which might function as an additional counteracting process responsible for the absent increase in NOS3 mRNA levels. Considering a recent report demonstrating eNOS uncoupling and decreased NO production with an enhanced superoxide production in adult rats after neonatal hyperoxia [32], the observed downregulation of NOS3 mRNA production in our study might be a protective mechanism downregulating eNOS expression upon oxygen exposure to prevent further superoxide production and tissue damage. Overall, the observed changes in epigenetic signatures are in line with recent reports such as the study by Zhu et al. who found that H3K27 trimethylation is present in a BPD mouse model and involved in RUNX3 downregulation, a gene associated with pulmonary development [33].

In the second part of the study, we provide further evidence for the relevance of the observed histone modifications for the regulation of NOS3 expression using an in vitro cell culture model. For correct interpretation of the data, it is important to mention that the physiological response pattern of human peripheral arterial endothelial cells is opposite to the response pattern of pulmonary endothelial artery cells, i.e., vasodilatation and NOS3 upregulation upon hypoxia and not hyperoxia. To induce a response pattern comparable to the mouse model upon hyperoxia, we therefore subjected HAEC to hypoxia after exposure to TSA. Similar to the mouse model, we observed long persistence of altered acetylation patterns after long-term exposure to TSA whereas histone signatures were restored after repetitive short-term exposure. In HAEC with long-term exposure to TSA, NOS3 mRNA levels remained high corresponding to an increase H3K9ac at the NOS3 gene locus. However, upon exposure to hypoxia, we observed a substantial decrease in NOS3 mRNA levels in these cells even though levels or H3K9ac further increased. Currently, similar to the constant increase in H3K27me3 at the NOS3 gene locus in these cells, we have no good explanation for this phenomenon. Possibly, this just reflects a complete disturbance and malfunction of the epigenetic code, but this needs to be further elucidated.

One major limitation of our study is the lack of functional studies such as measurement of NO. Since many previous studies have already demonstrated that upregulation of NOS3 corresponds very well with increased NO production in the hyperoxia mouse model [7], we believe that mRNA levels are sufficient physiological to characterize the cellular response patterns. In addition, even if that is not the case, it does not change the disturbances observed on the level of mRNA expression and in epigenetic signatures. The second major limitation of this study is the lack of further functional analysis on the role of miRNA on the development of BPD. However, since this seems to be rather complex [34] and considering the focused rather than generalistic approach of this study, this would have been beyond the primary scope of this study.

## Conclusions

This study provides further evidence for the long-term relevance of early life events on changes in epigenetic signatures using a newborn mouse model of hyperoxia-induced BPD. It demonstrates the occurrence and relevance of persisting epigenetic marks for regulation of vascular endothelial response patterns upon physiological stimuli later in life. Even though currently very theoretical, this suggests that treatment of BPD and its sequelae by modification of epigenetic mechanisms might at least be an option.

## Abbreviations

BPD: Bronchopulmonary dysplasia
BW: Birth weight
eNOS: Endothelial nitric oxide synthase
GA: Gestational age
HAEC: Human arterial endothelial cell
HDAC: Histone deacetylase
HOX: Hyperoxia
NO: Nitric oxide
NOX: Normoxia
PCR: Polymerase chain reaction
TSA: Trichostatin A
